# Characterization of Inherently Chiral Electrosynthesized Oligomeric Films by Voltammetry and Scanning Electrochemical Microscopy (SECM)

**DOI:** 10.3390/molecules25225368

**Published:** 2020-11-17

**Authors:** Margherita Donnici, Rosanna Toniolo, Serena Arnaboldi, Patrizia R. Mussini, Tiziana Benincori, Roberto Cirilli, Salvatore Daniele

**Affiliations:** 1 Dipartimento di Scienze Molecolari e Nanosistemi, Università Ca’ Foscari Venezia, Via Torino 155, 30172 Mestre-Venezia, Italy; margherita.donnici@unive.it; 2 Dipartimento di Scienze Agroalimentari, Ambientali e Animali, Università di Udine, Via Cotonificio 108, 33100 Udine, Italy; rosanna.toniolo@uniud.it; 3 Dipartimento di Chimica, Università degli Studi di Milano, 20133 Milano, Italy; serena.arnaboldi@unimi.it (S.A.); patrizia.mussini@unimi.it (P.R.M.); 4 Dipartimento di Scienza ed Alta Tecnologia, Università degli Studi dell’Insubria, 22100 Como, Italy; tiziana.benincori@uninsubria.it; 5 Centro Nazionale per il Controllo e la Valutazione dei Farmaci, Istituto Superiore di Sanità, 00161 Roma, Italy; roberto.cirilli@iss.it

**Keywords:** Inherently chiral oligomer films, chiral electroactive probes, scanning electrochemical microscopy, enantiodiscrimination in electrochemistry and electroanalysis

## Abstract

A voltammetric and scanning electrochemical microscopy (SECM) investigation was performed on an inherently chiral oligomer-coated gold electrode to establish its general properties (i.e., conductivity and topography), as well as its ability to discriminate chiral electroactive probe molecules. The electroactive monomer (*S*)-2,2′-bis(2,2′-bithiophene-5-yl)-3,3′-bibenzothiophene ((*S*)-BT_2_T_4_) was employed as reagent to electrodeposit, by cyclic voltammetry, the inherently chiral oligomer film of (*S*)-BT_2_T_4_ (oligo-(*S*)-BT_2_T_4_) onto the Au electrode surface (resulting in oligo-(*S*)-BT_2_T_4_-Au). SECM measurements, performed in either feedback or competition mode, using the redox mediators [Fe(CN)_6_]^4−^ and [Fe(CN)_6_]^3−^ in aqueous solutions, and ferrocene (Fc), (*S*)-FcEA, (*R*)-FcEA and *rac-*FcEA (FcEA is *N*,*N*-dimethyl-1-ferrocenylethylamine) in CH_3_CN solutions, indicated that the oligomer film, as produced, was uncharged. The use of [Fe(CN)_6_]^3−^ allowed establishing that the oligomer film behaved as a porous insulating membrane, presenting a rather rough surface. This was inferred from both the approach curves and linear and bidimensional SECM scans, which displayed negative feedback effects. The oligomer film acquired semiconducting or fully conducting properties when the Au electrode was biased at potential more positive than 0.6 V vs. Ag|AgCl|KCl. Under the latter conditions, the approach curves displayed positive feedback effects. SECM measurements, performed in competition mode, allowed verifying the discriminating ability of the oligo-(*S*)-BT_2_T_4_ film towards the (*S*)-FcEA and (*R*)-FcEA redox mediators, which confirmed the results obtained by cyclic voltammetry. SECM linear scans indicated that the enantiomeric discriminating ability of the oligo-(*S*)-BT_2_T_4_ was even across its entire surface.

## 1. Introduction

**Scheme 1:**
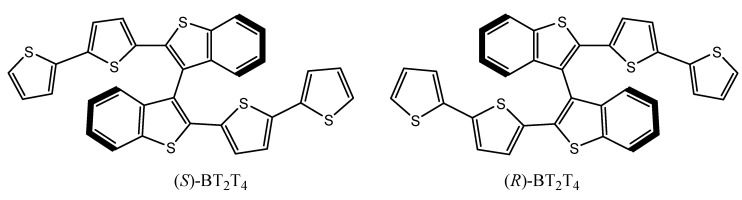
The (*S*)- and (*R*)-enantiomers of the inherently chiral BT_2_T_4_ monomer.

Conducting polymers (CPs) or oligomers consist of repeating units of conjugated electroactive organic molecules and display high chemical stability and conductivity [1]. They can mediate reactions while in their conductive state, making them viable as electrode materials. In addition, CPs are widely used as electrode modifiers to fine-tune the performance and quality of a variety of substrates [2]. In this regard, considerable attention has been devoted to the preparation of CP-modified electrodes to be employed as chemo- and bio-sensors to enhance sensitivity and selectivity in electroanalytical applications [2,3,4]. Among other desired sensing properties, enantiomer discrimination and quantification are fundamental for chiral compounds of interest in pharmaceutical, food, fragrance and environmental fields. Chirality is often introduced into CPs by attaching chiral pendants to the monomers to be (electro)oligomerized to yield the CP conjugated backbone [5], but other alternative approaches have also been proposed [6]. Outstanding chirality properties have been recently observed with electroactive films electrosynthesized from “inherently chiral” electroactive monomers, designed so that chirality and electroactivity originate from the same element, coinciding with the main molecular backbone featuring a tailored torsion on account of an atropisomeric or helical building block with associated high racemization barrier; this results in powerful chirality manifestations, possibly also electrochemically modulated [6]. The so far best studied example of inherently chiral monomer is 2,2′-bis(2,2′-bithiophene-5-yl)-3,3′-bibenzothiophene (BT_2_T_4_) (Scheme 1), featuring a 3,3′-bibenzothiophene *atropisomeric* core (with sterically hindered rotation between its two halves); its *C*_2_ symmetry results in propagation and amplification of its outstanding chirality properties in macro- and supramolecular structures [7].

Because these monomers are electroactive and have high electrooligomerization ability, they represent suitable starting reagents for the electrodeposition of inherently chiral oligomers onto electrode surfaces [6,7,8]. Chemically produced oligomers can also be dropcasted onto the electrode surface [9]. These oligomer-modified electrodes have displayed outstanding enantiodiscrimination ability for chiral electroactive probes in terms of large potential differences in voltammetric experiments [6,7,9,10,11,12].

In previous works, the electrochemical characterization of these electrodes has been performed considering voltammetry, spectroelectrochemistry and in-situ conductance techniques [6,7,8,9,10,11,12,13]. These techniques normally provide information on the whole and bulk material. Further knowledge of the chemical and electrochemical phenomena involved in such systems can be obtained by high spatial resolution electrochemical techniques, such as scanning electrochemical microscopy (SECM) [14]. SECM is a scanning probe technique based on the amperometric signal generated at a microelectrode [15] by a redox-active species in solution (redox mediator), which can perform quantitative local electrochemical experiments for studying heterogeneous and homogeneous reactions and for high-resolution imaging of the chemical reactivity and topography of various surfaces [14,15,16,17].

In the context of conducting polymers, SECM has been used to characterize poly(alkylterthiophene) films [18] and polypyrrole [19], to establish semiconducting surfaces and to study lateral charge transfer inside molecular films [20]. To the best of our knowledge, no SECM study exists dealing with the characterization of chiral polymers or oligomers.

In this work, for the first time, we report a SECM study to characterize the enantiopure (*S*)-BT_2_T_4_ oligomer, electrodeposited onto the surface of a gold disk electrode, starting from the corresponding (*S*)-BT_2_T_4_ monomer. In particular, the study aims at establishing whether: (i) The oligomer film acts as a porous membrane, with resulting electron transfer occurring at the underlying metal electrode; (ii) the electron transfer is localized at the oligomer/solution interface with the conducting oligomer film behaving as a conductive electrode material; (iii) a catalytic electron-transfer process occurs between the conducting polymer and a redox couple present in the bathing solution. In addition, the investigation aims at establishing how the chiral properties of the film affects the redox process of chiral compounds. To this purpose, voltammetric and SECM measurements were performed using different redox mediators charged and uncharged, without and with chiral properties.

## 2. Results and Discussion

### 2.1. Electrosynthesis and Voltammetric Characterization of the Oligomeric BT_2_T_4_ Film

**Figure 1:**
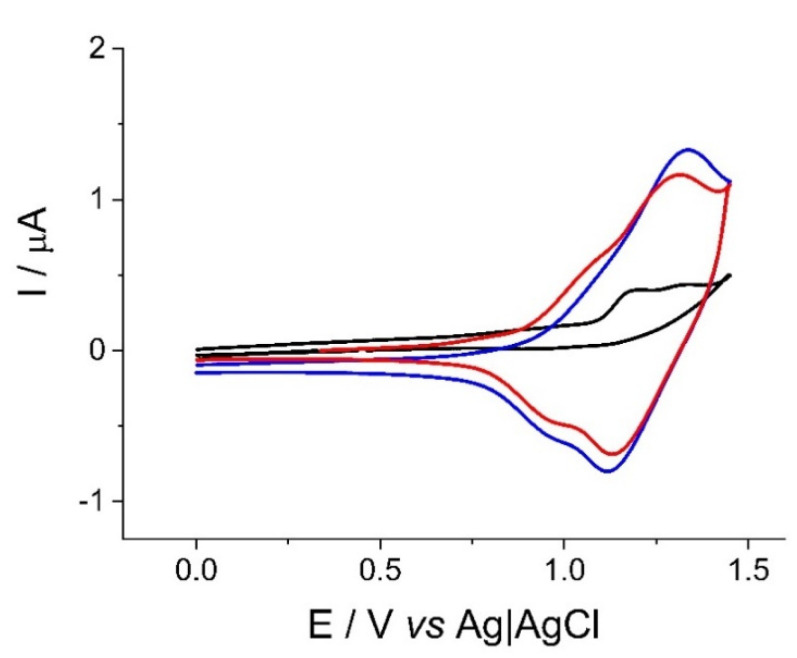
Cyclic voltammograms (CVs) recorded in a 0.5 mM (*S*)-BT_2_T_4_ CH_3_CN solution + 0.1 M TBAPF_6_ at Au disk electrode: First cycle (black line); fortieth cycle (red line). CV recorded at the oligomer-modified Au disk electrode in a (*S*)-BT_2_T_4_-free CH_3_CN solution + 0.1 M TBAPF_6_ (blue line). Scan rate 0.2 Vs^−1^.

The chiral oligomer film was electrodeposited onto a polycrystalline gold disk surface, starting from the enantiopure (*S*)-BT_2_T_4_ monomer and using a protocol analogous to those reported in detail elsewhere [8,10,11,12]. In brief, repeated oxidative potential cycling at 0.2 V s^−1^ was performed over the potential region 0–1.4 V vs. Ag/AgCl (KCl, saturated), in a CH_3_CN solution containing 0.5 mM (*S*)-BT_2_T_4_ and 0.1 M TBAPF_6_, as supporting electrolyte. The first and fortieth cycles are displayed in Figure 1 (with black and red line, respectively).

In the first cycle a two-peak process was observed, the first of which is due to the oxidation of the terthiophene moiety; the second corresponds to a further oxidation process of an electroactive product formed by a chemical follow up reaction, since it disappears at increasing scan rates (instead in less polar CH_2_Cl_2_, a first oxidation system is observed consisting of two nearly merging peaks accounting for the two molecule moieties behaving as interacting equivalent redox centres [7]). In the second and following cycles, a shift of the first oxidation peak at less positive potential and an overall current increase were observed. The potential shift points to an increased effective conjugation as a consequence of the coupling of terthiophene π systems, resulting in electroactivity features close to a linear hexathiophene [21]. The current increase, by increasing the number of cyclic voltammetric cycles, together with the presence of ill-defined cathodic peaks, recorded upon potential scan reversal, indicate the deposition of a solid, electrochemically active product on the electrode surface. According to earlier works, the deposited material is made of oligomers, from open and cyclic dimers to higher terms, with dimers largely prevailing in high-resolution laser desorption ionization (HR LDI) spectra [7,8,9]. The stability of the deposited film was verified by performing CV measurements with gold-modified electrode in a (*S*)-BT_2_T_4_-free acetonitrile solution and in the same potential window. The CV thus recorded displayed, essentially, the same features of that obtained in the medium containing the monomer (Figure 1, blue line). The presence of the oligomer onto the electrode surface was also verified by SEM and relevant EDX analysis (Supporting Materials S1).

**Figure 2:**
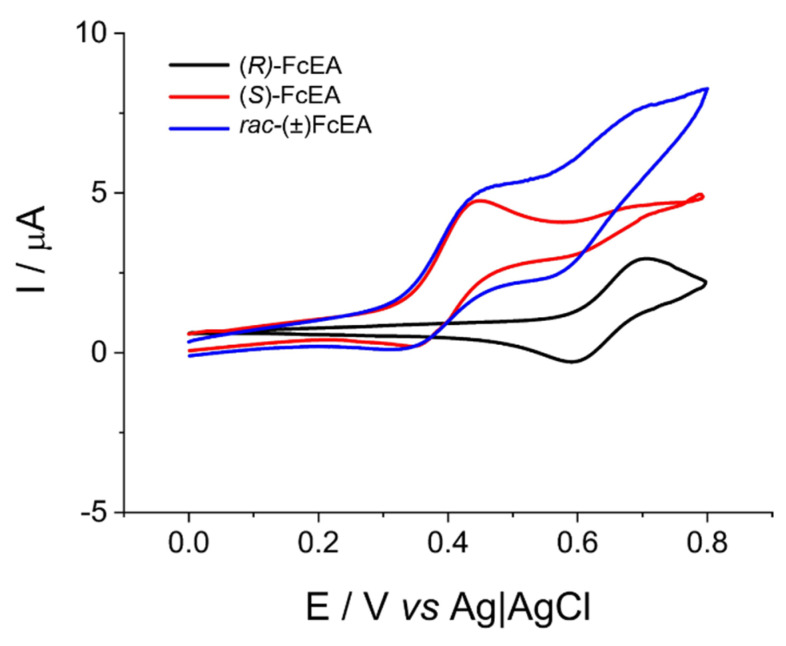
CVs recorded at an oligo-(*S*)-BT_2_T_4_- Au electrode in a CH_3_CN + 0.1 M TBAPF_6_ solution containing 1 mM (*S*)-FcEA, 2 mM *rac*-FcEA and 0.6 mM (*R*)-FcEA (as indicated in the figure). Scan rate 0.05 V s^−1^.

The enantio-recognition ability of the oligomer-modified gold electrode (oligo-(*S*)-BT_2_T_4_-Au) was assessed by CV in acetonitrile solutions containing the chiral probes (*S*)-(−), (*R*)-(+) and racemate *rac*-(±) of *N*,*N*-dimethyl-1-ferrocenylethylamine (FcEA) ((*S*)-FcEA, (*R*)-FcEA and *rac*-(±)FcEA, respectively), as shown in Figure 2. For comparison, the CVs of the same probes were performed using the bare Au-disk electrode (Figure S2).

It is seen that at the oligo-(*S*)-BT_2_T_4_-Au a clear separation of the half-wave potentials (*E*_1/2_) (obtained by the half sum of the anodic (*E*_p,a_) and cathodic (*E*_p_,_c_) peak potentials, *E*_1/2_ = (*E*_pc_ + *E*_pa_)/2 [15], of about 240 mV exists between the (*S*)-FcEA and (*R*)-FcEA probes when present in solution either alone or in the racemic mixture. It must be outlined that no such potential separation was observed at the naked Au electrode, where *E*_1/2_ values are identical (within ± 5 mV, experimental error) regardless of the probe enantiomer configuration (Figure S2). Thus, as in previous cases [7,10], the combination (*S*)-oligoBT_2_T_4_ + (*S*)-FcEA (or specularly (*R*)+(*R*) [7,10]) results in a less positive potential respect to (*S*)-oligoBT_2_T_4_ + (*R*)-FcEA (or, specularly, (*S*)+(*R*) [7,10]). It is however worthwhile recalling that no particular meaning should be given to such absolute configuration combinations, since the (*R*) and (*S*) stereodescriptors are assigned according to rules with no direct link to physical properties [22].

### 2.2. SECM Characterization of the Bare Au Surface and Oligo-(S)-BT_2_T_4_-Au Substrate

#### 2.2.1. General Aspects

SECM measurements were performed using platinum microdisk electrodes of 12.5 µm radius with *RG* varying between 5 and 8 (SECM tips, see experimental for details). The redox mediators employed were: [Fe(CN)_6_]^4−^, [Fe(CN)_6_]^3−^ in aqueous solutions containing 0.1 M KCl; ferrocene (Fc) and the chiral (*S*)-FcEA, (*R*)-FcEA and *rac-*FcEA molecules in CH_3_CN solution containing 0.1 M TBAPF_6_. Steady-state voltammograms obtained at the platinum microelectrode for all redox mediators employed are shown in Figure S3. Relevant *E*_1/2_ are summarized in Table S1.

**Figure 3:**
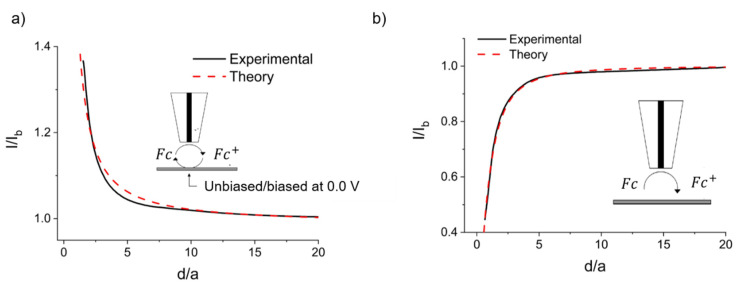
Approach curves recorded using Fc as redox mediator with a Pt microdisk 12.5 µm radius, above the bare gold surface (**a**) and the surrounding PTFE insulator (**b**). Red dashed lines refer to theoretical diffusion-controlled processes. The tip was biased at 0.4 V and the substrate was either unbiased or biased at 0.0 V.

Preliminarily, the surface status of the bare gold substrate (1 mm radius) for the electrodeposition of the oligo-(*S*)-BT_2_T_4_ film, was investigated by SECM in feedback mode [14]. In these experiments, the microelectrode tip was always biased at a potential at which the steady-state diffusion limiting current of each redox mediator was achieved. Instead, the substrate was either unbiased or biased at a potential at which the inverse of the reaction occurring at the SECM tip took place (see Scheme in Figure S4). Since in the case of the bare Au electrode, all redox mediators provided similar responses, in what follows, the results obtained for the redox system Fc^+^/Fc are discussed. Typical normalized approach curves, i.e., normalized current, (*I/I*_b_) (*I* is the current at given tip-to-substrate distance *d*, *I*_b_ is the current in the bulk solution), against normalized distance (*d/a*) (*a* is the radius of microelectrode) obtained above the active metal surface and, for comparison, above the surrounding insulating material, are shown in Figure 3. Above the Au surface, regardless of whether the substrate was biased or not, a positive feedback current (i.e., current increases as the tip-to substrate distance decreases), overlapping the theoretical curve for a diffusion-controlled process, was obtained (Figure 3a). This accords with the regeneration mechanism of the redox mediator characteristic for a conductive substrate (Scheme in Figure S4 and in inset in Figure 3a) [14,23,24,25,26,27,28]. The positive feedback response, also obtained at the unbiased substrate, is due to the phenomenon of the lateral electron transfer that occurs when the substrate area, bathed by the redox mediator solution, is much larger than that of the microelectrode [14,23,24,25,26,27,28], as is the present case (see comments in Figure S4’s caption for details). Above the insulator, the approach curves provided negative feedback (i.e., current decreases as the tip-to substrate distance decreases) overlapping that for a diffusion-controlled process. This behavior is related to the hindered diffusion of the redox mediator towards the microelectrode surface (see inset in Figure 3b). Approach curves like those described above were also recorded by performing measurements above different locations of both Au disk and insulator.

**Figure 4:**
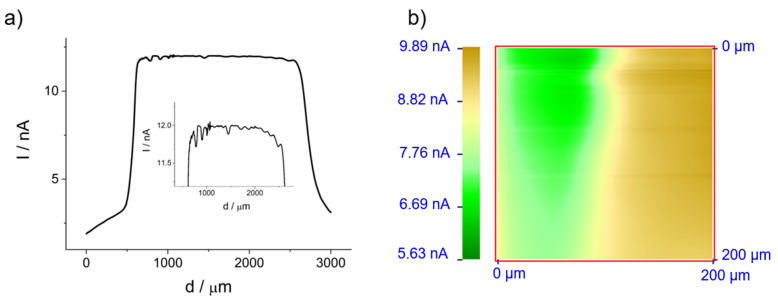
One-dimensional (**a**) and bi-dimensional (**b**) scans obtained by using Fc as redox mediator and a Pt microdisk with 12.5 µm radius. The tip was positioned at 10 µm above the substrate.

The uniformity of the responses across the substrate was also assessed by performing one-dimensional (1D) and bi-dimensional (2D) scans. In these experiments, the microelectrode was initially located at 10 µm above the substrate and the scans were started with the microelectrode positioned above the insulator. Then, it was moved towards the active metal surface. Typical 1D and 2D scans are shown in Figure 4. Insulating (or not active) and conducting (or active) materials correspond to the zones with lower and higher currents, respectively. The abrupt current changes, from low to high values (or vice-versa in Figure 4a), indicate the transition from the insulating to the conducting zone of the material (or vice-versa). Current spikes and oscillations over the active zone (see inset in Figure 4a) reflect, conceivably, the high roughness of the substrate. An estimate of the roughness of the bare Au surface was made by using the arithmetic mean value of the current oscillations obtained in the 1D scans and using the theoretical approach curves, to transform current values in distances (Supporting Materials, S5). In particular, the roughness average (*Ra*) was 1.5 µm, while maximum peak to valley height (*R_max_*) was 2.6 µm.

Series of SECM measurements, as those described above, were performed using the oligo-(*S*)-BT_2_T_4_-Au substrate in feedback and competition mode; in the latter case, the applied potentials at both SECM tip and substrate were such that the same process of the redox mediator (oxidation or reduction) occurred. In addition, to differentiate between general (conductivity, permeability and topography) and enantiomeric properties of the oligo-(*S*)-BT_2_T_4_ film, the results obtained with the conventional (i.e., achiral) and chiral redox mediators are discussed separately.

#### 2.2.2. Achiral Redox Mediators

**Figure 5:**
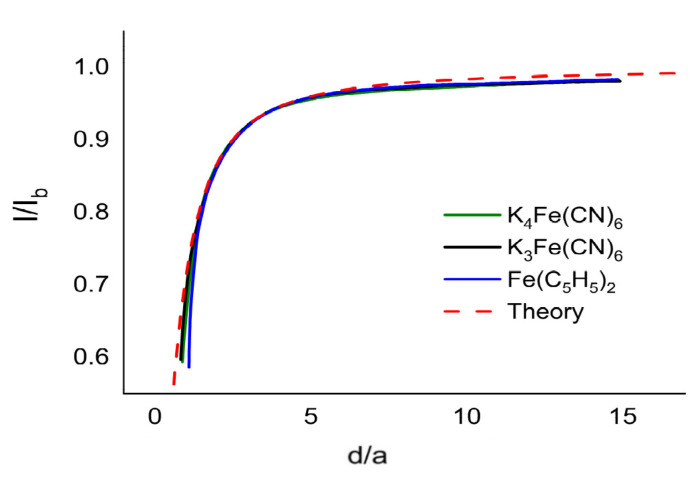
Approach curves recorded with the indicated redox mediator above the unbiased oligo-(*S*)-BT_2_T_4_-Au. The scheme shown in the inset represents, as an example, the process occurring using Fc as redox mediator.

Typical approach curves obtained at the unbiased oligo-(*S*)-BT_2_T_4_-Au are shown in Figure 5. As is evident, all redox mediators provide *I/I*_b_ vs. *d/a* profiles that fit the theory for negative feedback responses. Comparative measurements, performed above the insulating material surrounding the Au surface, gave similar responses (not shown). These results suggest that, under the above conditions, the oligomer film acts as an insulating membrane and that no ion exchange process takes place between ion species, eventually trapped within the oligomer, and the charged redox mediators in solution. This can be because the oligomer film was in its uncharged state. On the other hand, based on the *E*_1/2_ values shown in Table S1, the above results are congruent with the circumstance that the various redox mediators were not able to locally inject or subtract electrons, i.e., to act as reductant or oxidant, towards the oligo-(*S*)-BT_2_T_4_-film, whose oxidation or reduction processes occur at higher (Figure 1) or much lower potentials [21]. The latter occurrence would have involved the regeneration of the redox mediator at the oligomer/solution interface, which would manifest through positive feedback currents. Permeation of the redox mediators within the oligomer film down to the underlying gold surface (whose open circuit potential would be defined by the solution redox species) cannot be excluded. However, even if this were the case, the *I/I*_b_ vs. *d/a* responses in Figure 5 would indicate that the local portion of the substrate surface bathed by the redox mediator solutions, and therefore participating in the feedback loop, was not large enough to support any substantial current enhancement [23,24,25,26,27,28,29]. It must be noticed that the lack of current enhancement could also be related to both the thickness of the oligomer film, which could be too thick for redox mediator regeneration at the Pt microdisk surface [18,19], and the actual closest tip- to-substrate distance, which could be achieved to avoid film penetration. As for the latter aspect, from the approach curve in Figure 5, and considering the *I/I*_b_ value of 0.4 (approximately corresponding to the closest *d/a* achieved while bringing the tip towards the substrate), an average tip to- substrate separation of about 8 µm was estimated. In fact, attempts to bring the SECM tip closer led to a flattening of the tip current, indicating that the tip came into contact with the oligomer film. Possible explanations for such behavior include a significant roughness of the substrate material and lack of precise perpendicular alignment of the tip above the substrate. Clearly, the above circumstances limited to some extent the resolution of the measurements and can in part explain the observed negative feedback effects.

**Figure 6:**
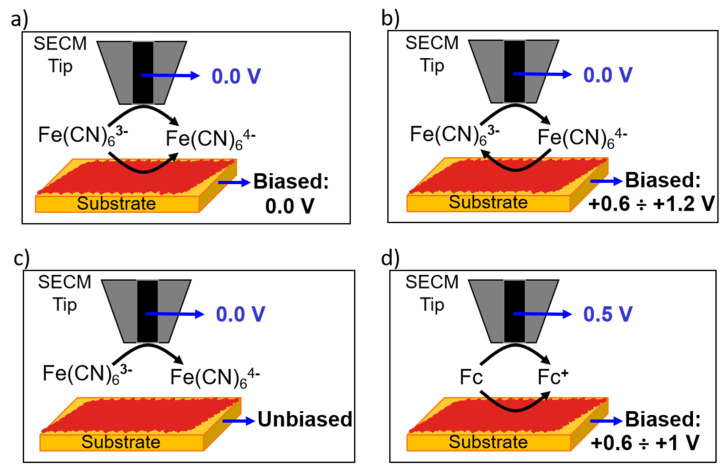
Schemes of the redox processes occurring at the microelectrode tip and substrate at different potentials applied at the substrate. (**a**–**c**) and (**d**) refer to Fe(CN)_6_^3−^ and Fc redox mediator, respectively. Potential applied to the tip and substrate as indicated in the figure.

The effect of the application of the potential to the oligo-(*S*)-BT_2_T_4_-Au was then investigated. The case of Fe(CN)_6_^3−^ redox mediator is discussed in detail as, unlike Fc and Fe(CN)_6_^4−^, at the microelectrode, a diffusion limiting current is achieved by applying a potential negative to its *E*_1/2_ value and the experiments provided somewhat different results. In the measurements, the microelectrode tip was biased at 0.0 V, at which the reduction of Fe(CN)_6_^3−^ to Fe(CN)_6_^4−^ is under diffusion control (see schemes in Figure 6a–c and Figure S3f). The substrate was biased at different potentials over the range 0–1.2 V at which either the competitive reduction (scheme in Figure 6a) or the regeneration (scheme in Figure 6b) of the redox mediator could occur.

**Figure 7:**
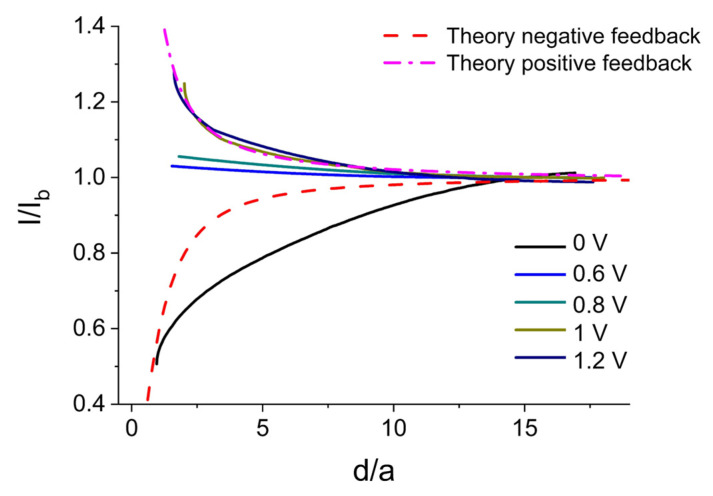
Approach curves recorded in a 2 mM K_3_Fe(CN)_6_ + 0.1 M KCl water solution with the microelectrode tip biased at 0.0 V, above the oligo-(*S*)-BT_2_T_4_-Au biased at different potentials as indicated.

Typical approach curves thus recorded are displayed in Figure 7.

When the substrate is biased at 0.0 V, the tip current decreases, starting far away from the substrate. This is because the redox mediator is reduced competitively at both microelectrode and substrate, as schematized in Figure 6a. The redox process at the substrate, conceivably, occurs at the underlying Au surface, through permeation of [Fe(CN)_6_]^3−^ within the film or through film defects and porosity. Therefore, the *I/I*_b_ vs. *d/a* profile follows the depletion of the concentration of Fe(CN)_6_^3−^ within the diffusion layer that grows in the solution above the substrate. At higher applied potentials, the *I/I*_b_ vs. *d/a* profile changes from negative into positive feedback, indicating the occurrence of the redox mediator regeneration, as schematized in Figure 6b. However, over the potential region 0.6–0.8 V, the current increase (Figure 7 with blue and green line) is below the diffusion-controlled process for positive feedback (Figure 7, magenta dashed line). This can be due to the fact that the location of the electron transfer can be at the underlying gold surface, which being coated by the oligomer film can work as a partially blocked electrode, at which the electron transfer process can be somewhat inhibited [23,30]. Notably, the problem of Au surface availability is here more important respect to the above case of competition mode, since to obtain positive feedback the mediator should both diffuse to the Au surface to be oxidized and retro-diffuse out of the porous layer to reach the tip. It is also worthwhile noting that, unlike in the above case of competition mode, in this case only the surface below the microelectrode tip is involved [14,23,28,29]. Moreover, over the latter potential range, the oligo-(*S*)-BT_2_T_4_ begins to be doped (see Figure 1), the film can acquire semiconductive properties and the process can occur at the oligomer/solution interface (or within the film). At potentials above 0.8 V, the conductivity of the oligomer remarkably increases [13], with the film behaving as a metal electrode. In fact, at 1.0 and 1.2 V, the experimental approach curves almost fit the theory. The positive feedback current can also be due to a catalytic electron-transfer process between the oxidized oligomer (reaction (1) and the reduced form of redox mediator (reaction (2)), formed at the microelectrode tip and diffusing towards the oligo-(*S*)-BT_2_T_4_ film:

oligo-(*S*)-BT_2_T_4_ ⇋ oligo-(*S*)-BT_2_T_4_^n+^ + ne^−^   (*n* = 1 or 2) (1)

oligo-(*S*)-BT_2_T_4_^+^ + [Fe(CN)_6_]^4−^ ⇋ oligo-(*S*)-BT_2_T_4_ + [Fe(CN)_6_]^3−^   (for *n* = 1) (2)

The latter sequence, however, would include, in step (1), an anion exchange process, in which the redox mediator itself might be involved although the supporting electrolyte anion is smaller and at higher concentration. This would limit the overall kinetic of the process.

**Figure 8:**
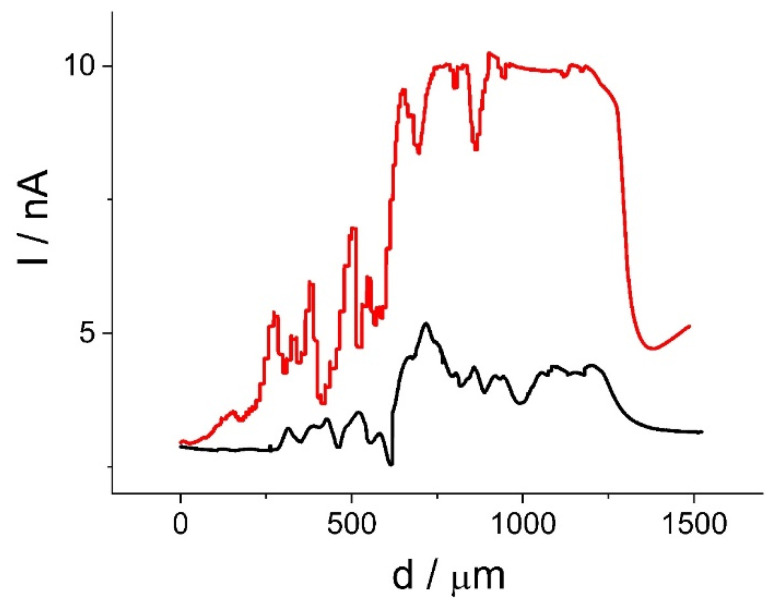
One-dimensional scans recorded in a 2 mM K_3_Fe(CN)_6_ + 0.1 M KCl water solution with the microelectrode tip located at 10 µm above the oligo-(*S*)-BT_2_T_4_-Au. Microelectrode tip biased at 0.0 V substrate unbiased (black line) and biased at 1 V (red line).

**Figure 9:**
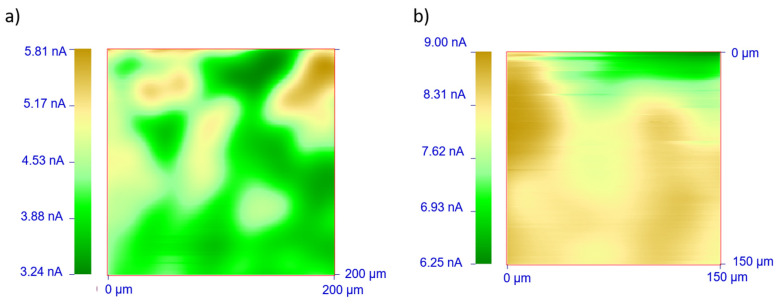
Bi-dimensional scans recorded in a 2 mM K_3_Fe(CN)_6_ + 0.1 M KCl water solution with the microelectrode tip located at 10 µm above the oligo-(*S*)-BT_2_T_4_-Au. Microelectrode tip biased at 0.0 V substrate unbiased (**a**) and biased at 1 V (**b**).

The uniformity of the properties of the oligo-(*S)*-BT_2_T_4_-Au was established by performing 1D and 2D scans across the substrate surface, using Fe(CN)_6_^3−^ as redox mediator. For the 1D scan, the microelectrode was initially located at 10 µm above the insulating zone and then moved towards the oligo-(*S*)-BT_2_T_4_-Au material. The 2D scans were instead made on a 150 µm × 150 µm square zone above the oligomeric film and at a tip-to substrate distance of 10 µm. The tip was always biased at 0.0 V, while the substrate was either unbiased or biased at 1.0 V. Under these conditions, the oligo-(*S*)-BT_2_T_4_-Au behaved as an insulating or an active material, respectively, at which the electrode processes schematized in Figure 6c,b respectively, occurred. Typical 1D and 2D images thus obtained are shown in Figure 8 and Figure 9.

The *I* vs. *d* profile in Figure 8 with the black line and the image in Figure 9a, both characterized by relatively low currents, reflect, essentially, the topography of the material; the current spikes in Figure 8 are conceivably due to the surface roughness (including defects in the film), which as mentioned above, seems to be rather high. The scan displayed with the red line in Figure 8 and the image in Figure 9b, characterized by larger currents, reflect both activity (i.e., conductivity) and topography of the oligo-(*S)*-BT_2_T_4_ film. The current spikes, again, are due to the rather rough topography of the investigated material. An estimate of the roughness of the oligo-(*S)*-BT_2_T_4_ film was made by the analysis of the current oscillations of the 1D scans, using the same approach employed above for the bare Au surface. *Ra and R*_max_ values were 2.1 and 4.1 µm, respectively, indicating a higher roughness with respect to the bare Au surface, as also appears from SEM images shown in Figure S1.

The above scenario concerning the general characteristics (conductivity and topography) of the oligomer film was also supported from SECM measurements performed with both Fc and Fe(CN)_6_^4−^ as redox mediators. In these experiments, the substrate was also kept either unbiased or biased over the same potential window as above. At the microelectrode tip, the potential applied was 0.55 and 0.4 V for Fc and Fe(CN)_6_^4−^, respectively, to achieve steady-state diffusion limiting currents. Again, with both redox mediators, a negative feedback current was recorded above the oligo-(*S*)-BT_2_T_4_-Au substrate (same as in Figure 5), kept either unbiased or biased at potentials less positive than the corresponding *E_1/2_* of the two redox mediators. This was due to the above mentioned less availability of the underlying gold surface and to the diffusion and retro-diffusion of the redox mediator couple through the oligomer film. Instead, a competitive oxidation process of the redox mediators occurred at both microelectrode tip and substrate (see, for example, scheme in Figure 6d for Fc), when the latter was biased at a potential more positive than the *E*_1/2_ values of Fc and Fe(CN)_6_^4−^. The *I/I*_b_ vs. *d/a* profiles (not shown for the specific cases) were as that displayed in Figure 7 with the black line. In fact, under the latter conditions, the oxidation of the two redox mediators occurs at both substrate (either at the underlying Au surface or at the oligomer/solution interface, the electroactive film being now conductive) and the microelectrode tip.

#### 2.2.3. Chiral Redox Mediators

**Figure 10:**
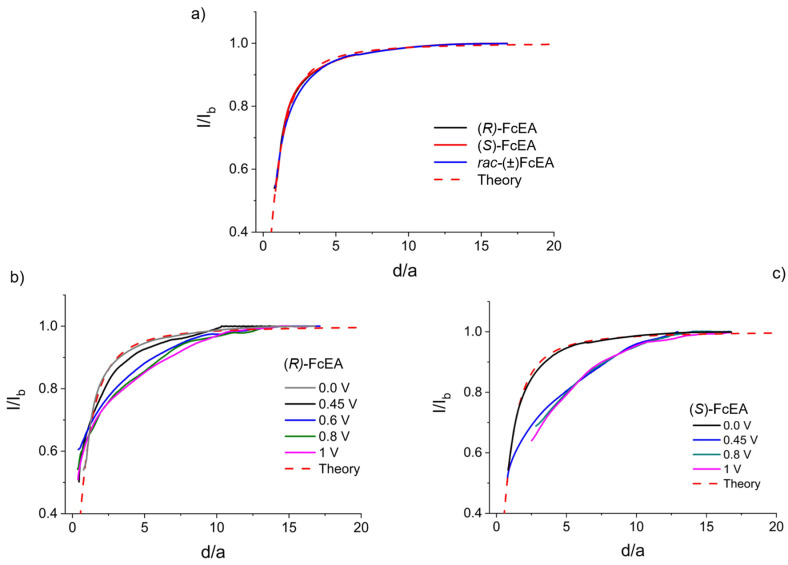
Approach curves recorded in 2 mM of (*S*)-FcEA or and 1 mM (*R*)-FcEA CH_3_CN solution with a Pt microdisk 12.5 µm radius, biased at 0.5 V, above the unbiased oligo-(*S*)-BT_2_T_4_-Au (**a**) and biased at different potentials (**b**,**c**) as indicated. Approach curves with dashed red lines refer to the theory for negative and positive feedback.

Figure 10 shows typical approach curves obtained with the different chiral redox mediators while the microelectrode tip was positioned above the unbiased (Figure 10a) and biased (Figure 10b,c) oligo-(*S*)-BT_2_T_4_-Au substrate.

Apparently, the results obtained by using the enantiomers of the chiral redox mediator are qualitatively similar to those described above for Fc. This is not surprising as, at the microelectrode tip, they share as expected the same *E*_1/2_ value (within experimental error, see Figure S3 and Table S1) and, as discussed above, the oligomer film behaved as an insulating porous membrane when it was in its uncharged form. Thus, at the unbiased substrate, negative feedback currents were recorded, regardless of the configuration of the chiral redox mediator (Figure 10a). Similarly, when the substrate was biased at 0.0 V, negative feedback currents were obtained (black lines in Figure 10b,c), due to both the limited active substrate area below the microelectrode tip and the relatively high *d/a* at which the microelectrode could be brought above the substrate (see above discussion). At higher applied potentials, the typical *I/I*_b_ vs. *d/a* responses for competitive oxidation of the redox mediators at both microelectrode tip and substrate occurred (blue, green and magenta lines in Figure 10b,c). However, in these cases, a careful analysis of the SECM responses allowed verifying that the decrease of *I/I*_b_, at same *d/a*, was larger for (*S*)-FcEA with respect to (*R*)-FcEA, indicating a clear discriminating ability of the oligo-(*S*)-BT_2_T_4_ film towards the two probe enantiomers. For instance, when the substrate was biased at 0.45 V, at which only (*S*)-FcEA could be oxidized (Figure 2), *I/I_b_* were about 0.65 and 0.77, at *d/a* = 2 and 4, respectively; the corresponding values (i.e., at same *d/a*) for (*R*)-FcEA were 0.78 and 0.92, respectively. As the substrate potential was shifted further towards more positive values (i.e., 1 V), the oxidation of both (*S*)-FcEA and (*R*)-FcEA was under diffusion control, and *I/I*_b_ vs. *d/a* acquired very close profiles.

**Figure 11:**
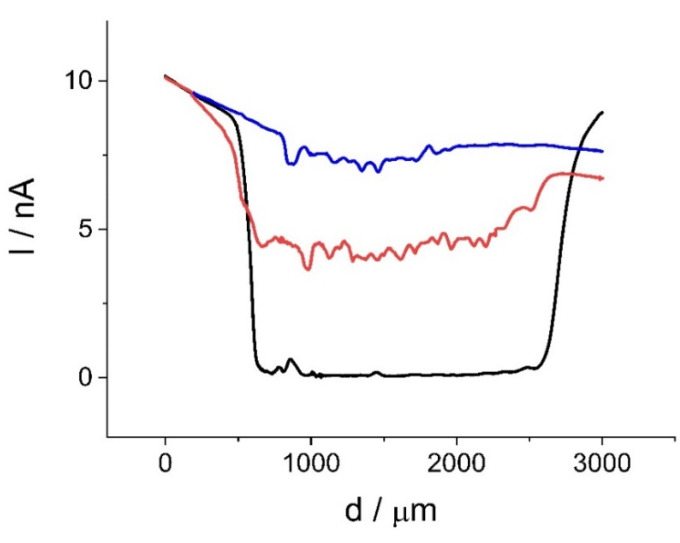
One-dimensional scans recoded recorded in 2 mM *rac-(±)*FcEA acetonitrile solution, with a Pt microdisk 12.5 µm radius, biased at 0.55 V, above the oligo(*S*)-BT_2_T_4_-Au: Unbiased (blue line), biased at 0.45 V (red line) and at 1 V (black line). Tip to substrate distance 10 µm.

To further support the above view, 1D scans were performed above the oligo-(S)-BT_2_T_4_-Au substrate using *rac*-(±)FcEA, as redox mediator (Figure 11). The microelectrode tip was biased at 0.55 V, while the substrate was either unbiased (blue line) or biased at 0.45 V (red line) and 1 V (black line). Again, the scans were started from the insulating portion of the substrate and the microtip moved towards the entire section of oligo-(*S*)-BT_2_T_4_-Au. At the unbiased substrate, current spikes, conceivably due to the substrate topography (see above discussion), were recorded. When the substrate was biased at 0.45 V only (*S*)-FcEA could be oxidized (Figure 2) and the current decreased to a significant extent, due to the consumption of the probe at the underlying gold surface. The current decrease was much more marked when the substrate was biased at 1 V, at which both (*S*)-FcEA and (*R*)-FcEA could be oxidized. These results therefore confirm the enantiodiscrimination ability of the oligo-(*S*)-BT_2_T_4_ film and that the phenomenon occurred, essentially, evenly across the entire substrate surface.

## 3. Materials and Methods

### 3.1. Chemicals

Acetonitrile (anhydrous, 99.9%), tetrabutylammonium hexafluorophosphate (TBAPF_6_), ferrocene (Fc), (*S*)-(−), (*R*)-(+) and racemate of *N*,*N*-dimethyl-1-ferrocenylethylamine (FcEA) ((*S*)-FcEA, (*R*)-FcEA and *rac-*FcEA), potassium ferro- and ferri-cyanide, potassium chloride, hexaammineruthenium(III) trichloride, were purchased from Sigma Aldrich (Aldrich, St. Louis, MO, USA) and used as received. The monomer (*S*)-BT_2_T_4_ was prepared, purified and resolved into antipodes as reported earlier [7]. The aqueous solutions were prepared with deionized water purified via a Milli-Q unit (Millipore system). When required pure nitrogen (99.99%, from SIAD, Bergamo, Italy) was used to de-aerate both acetonitrile and the aqueous solutions.

### 3.2. Apparatus and Electrodes

A CHI920B workstation (CH Instruments, Austin, TX USA) was employed for both voltammetric and SECM measurements, and unless otherwise stated all experiments were performed in an electrochemical cell in a three-electrode configuration. For the voltammetric characterization of the of the redox probes/mediators and the electrosynthesis of the oligo-(*S*)-BT_2_T_4_ film, a polycrystalline gold disk 2 mm diameter (from Amel, Milano, Italy) embedded in a PTFE rod, was employed as working electrode; a platinum spiral and an Ag/AgCl (KCl, saturated) were employed as counter and reference electrode, respectively. For the measurements performed in acetonitrile, the reference electrode was placed in a compartment ending in a porous frit, filled with the working solvent and supporting electrolyte, to avoid contamination of the working solution with water and chloride ions. The Au disk, prior use, was mechanically polished with a diamond suspension (0.1 µm diameter) placed over a Buehler microcloth, and then rinsed with milli-Q water and acetonitrile.

Platinum disk microelectrodes with nominal radius of 12.5 µm were employed as tip/working electrodes in either SECM or voltammetric measurements to characterize the redox mediators by steady-state voltammetry, and the local activity of the oligo-(*S*)-BT_2_T_4_-Au material. The microelectrodes were prepared by sealing platinum wires (Goodfellow Metals, Cambridge, UK) within glass capillaries, following a standard procedure [24]. Afterward, they were tapered to a conical shape, polished with graded alumina powder (5, 1 and 0.3 µm), placed on a Buehler microcloth and then characterized by cyclic voltammetry at low scan rates and by SECM to evaluate the actual electrode radius of the microelectrode and the overall tip radius to the electrode radius ratio (*RG*) [14]. The geometric radius of the microdisk was calibrated by recording the steady-state diffusion limiting current (*I_b_*) from a 1 mM Ru(NH_3_)_6_Cl_3_ solution containing 0.1M KCl and using the following equation [31]:

*I_b_* = *4 n F D c^b^ a* (3)

where *n* is the number of electrons, *F* is the Faraday constant, *D* is the diffusion coefficient of the electroactive species (in this case 7.0 × 10^−6^ cm^2^s^−1^ [32]), *c^b^* is the bulk concentration and *a* is the radius of the microdisk. The *RG* parameter was evaluated by fitting fitting experimental approach curves recorded under purely diffusion-controlled conditions to the theoretical curves [33,34]. The *RG* thus estimated varied between 5 and 8.

In all SECM and voltammetric measurements, the reference/counter electrode was a Ag/AgCl (in saturated KCl); a platinum wire acted as counter electrode. Unless otherwise stated, approach curves were plotted using normalized currents, *I*/*I_b_*, against normalized distances *d*/*a*.

## 4. Conclusions

The voltammetric and SECM behaviour of the chiral (*S)*-BT_2_T_4_ oligomer, electrodeposited onto the surface of a gold disk electrode, was investigated by using a range of redox mediators with different characteristics (i.e., charged and uncharged, racemate and enantiopure antipodes). The results obtained with all achiral redox mediators have indicated that the oligomeric film, as produced, was uncharged. Using [Fe(CN)_6_]^3−^ in the SECM experiments allowed establishing the local properties of the fabricated oligomeric film in terms of topography and conductivity. Thus, it has been verified that the oligomeric film behaved as a porous insulating membrane, presenting a rather rough surface exposed to the bathing solution, when the substrate was biased at potentials below about 0.6 V vs. Ag/AgCl. This was inferred from the approach curves, which have displayed essentially negative feedback effects. The film acquired semiconducting properties over the potential region 0.6–0.8 V vs. Ag/AgCl, while it became fully conductive at 1.0 and 1.2 V vs. Ag/AgCl. Under these conditions, the approach curves have displayed positive feedback effects. In addition, SECM measurements, performed in competition mode, using chiral probes, have allowed assessing the discriminating ability of the oligo-(*S*)-BT_2_T_4_ film towards the (*S*)-FcEA and (*R*)-FcEA redox mediators, thus confirming results obtained by cyclic voltammetry. Thanks to the high spatial resolution of SECM, it has also been shown that the enantiomeric discriminating ability of the oligo-(*S*)-BT_2_T_4_ occurred evenly across the entire substrate surface.

Improvements in the spatial resolution of the SECM measurements can be obtained by using smaller microelectrode tips (e.g., at sub-micrometer levels). However, this would also require the use of smoother oligomer films, which in principle can be obtained by decreasing the number of CV cycles during the electrodeposition step and smoother surfaces of the underlying electrode material. These aspects will be addressed in future investigations.

## Supplementary Materials

The following are available online, Figure S1: SEM Images of the oligomer modified gold electrode and elemental analysis. Figure S2: cyclic voltammograms recorded at the bare Au (2 mm) dimeter electrode in CH_3_CN solutions containing Fc, (*S*)-FcEA, (*R*)-FcEA and *rac(±)*-FcEA. Figure S3: steady-state voltammograms recorded at the Pt microdisk 25 µm diameter in solutions containing all redox mediators employed in this work. Figure S4: Scheme of the redox mediator regeneration also due to lateral electron transfer occurring in the SECM experiments. S5: Method employed to evaluate the roughness of the bare Au and(*S)*-BT_2_T_4_ oligomer.
