# Supplementary material for: Characterization of Inherently Chiral Electrosynthesized Oligomeric Films by Voltammetry and Scanning Electrochemical Microscopy (SECM)
Source: Molecules. 2020 Nov 17;25(22):5368. doi: 10.3390/molecules25225368 (PMC7698396; doi:10.3390/molecules25225368)
Supplement: Supplementary file 1 [file molecules-25-05368-s001.pdf]

Supporting Materials

Characterization of Inherently Chiral Electrosynthesized Oligomeric Films by Voltammetry and Scanning Electrochemical Microscopy (SECM)

Margherita Donnici,[1] Rosanna Toniolo,[2] Serena Arnaboldi[3], Patrizia Mussini[3], Tiziana Benincori[4], Roberto Cirilli[5], Salvatore Daniele[1*]

Margherita Donnici [1], Rosanna Toniolo [2], Serena Arnaboldi [3], Patrizia R. Mussini [3], Tiziana Benincori [4], Roberto Cirilli [5] and Salvatore Daniele [1,*]

1. Dipartimento di Scienze Molecolari e Nanosistemi, Università Ca' Foscari Venezia, Via Torino 155, 30172 Mestre-Venezia, Italy; margherita.donnici@unive.it
2. Dipartimento di Scienze Agroalimentari, Ambientali e Animali, Università di Udine, Via Cotonificio 108, I-33100 Udine, Italy; rosanna.toniolo@uniud.it
3. Dipartimento di Chimica, Università degli Studi di Milano, 20133 Milano, Italy; serena.arnaboldi@unimi.it (S.A.); patrizia.mussini@unimi.it (P.R.M.)
4. Dipartimento di Scienza ed Alta Tecnologia, Università degli Studi dell'Insubria, 22100 Como, Italy; tiziana.benincori@uninsubria.it
5. Centro Nazionale per il Controllo e la Valutazione dei Farmaci, Istituto Superiore di Sanità, 00161 Roma, Italy; roberto.cirilli@iss.it

* Correspondence: sig@unive.it; Tel.: +39-041-23448630

Corresponding author:

Prof. Salvatore Daniele

Tel. +39 041 23448630

Fax: +39 041 23448595

e-mail: sig@unive.it

Figure S1

SEM Images of the bare (A) and oligomer-modified (B) polycrystalline gold electrode

A)

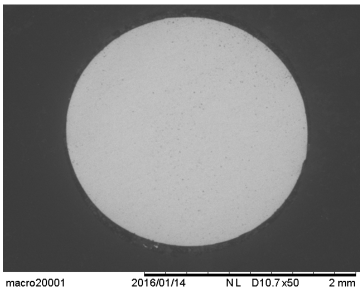

B)

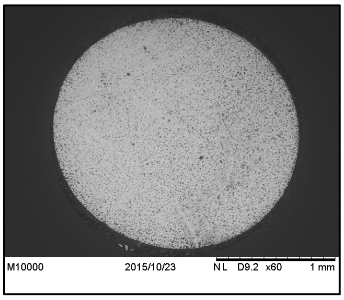

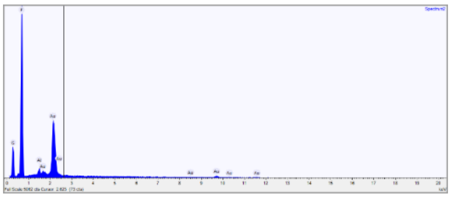

EDX analysis of the surface

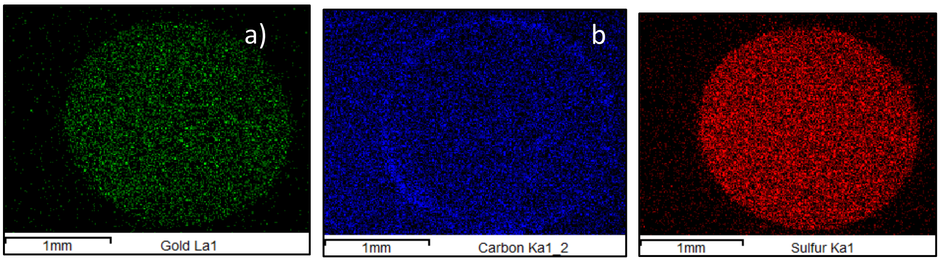

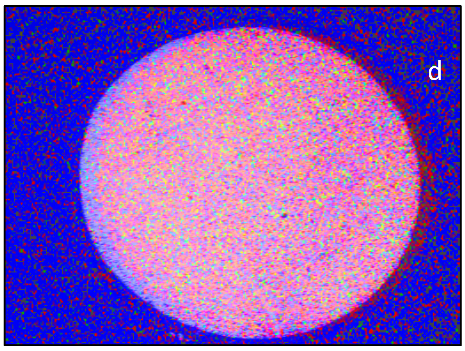

1mm Mixed

Elemental analysis: a) gold (green), b) carbon (blue), c) sulfur (red), d) mixed elements.

Figure S2- Cyclic voltammograms recorded at the bare gold electrode (2 mm diameter) in a CH_3CN + 0.1 M $TBAPF_6$ solution containing 1 mM each of Fc (red line), (*S*)-FcEA (black line), (*R*)-FcEA (green line) and *rac*(±)-FcEA (blue line) probes. Scan rate 0.050 V s^{-1}

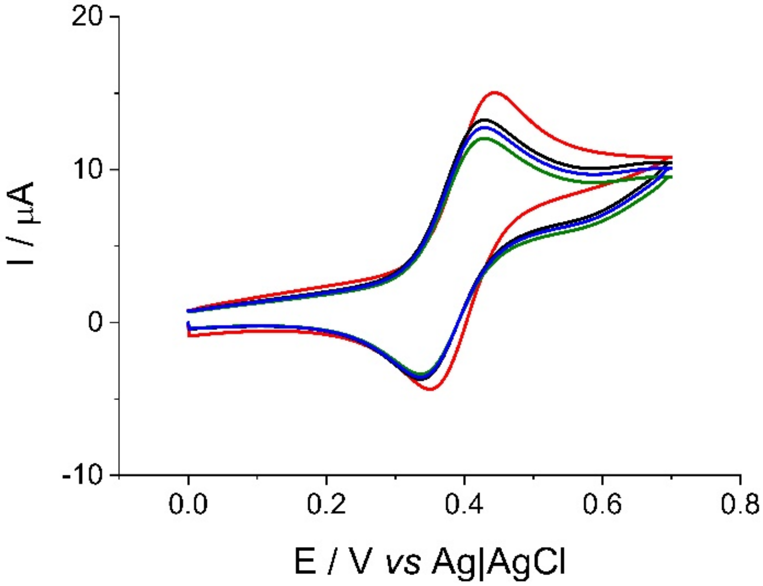

.

Figure S3- Cyclic voltammograms recorded at 0.005V s^{-1} with a Pt microdisk electrode (25 μm diameter) in the following media: CH_3CN + 0.1 M $TBAPF_6$ solution containing 1 mM Fc (a), 0.6 mM (*R*)-FcEA (b), 1 mM (*S*)-FcEA (c), 1 mM *rac*(±)-FcEA (d) redox mediators; H_20 + 0.1 M KCl solutions containing 1 mM $K_4[FeCN)_6]$ (e), 1 mM $K_3[FeCN)_6]$ (f) redox mediators. Acronyms Fc and FcEA stand for ferrocene and of *N*,*N*-dimethyl-1-ferrocenylethylamine, respectively. *rac*- stands for racemate of the *S*- and *R*- enantiomers.

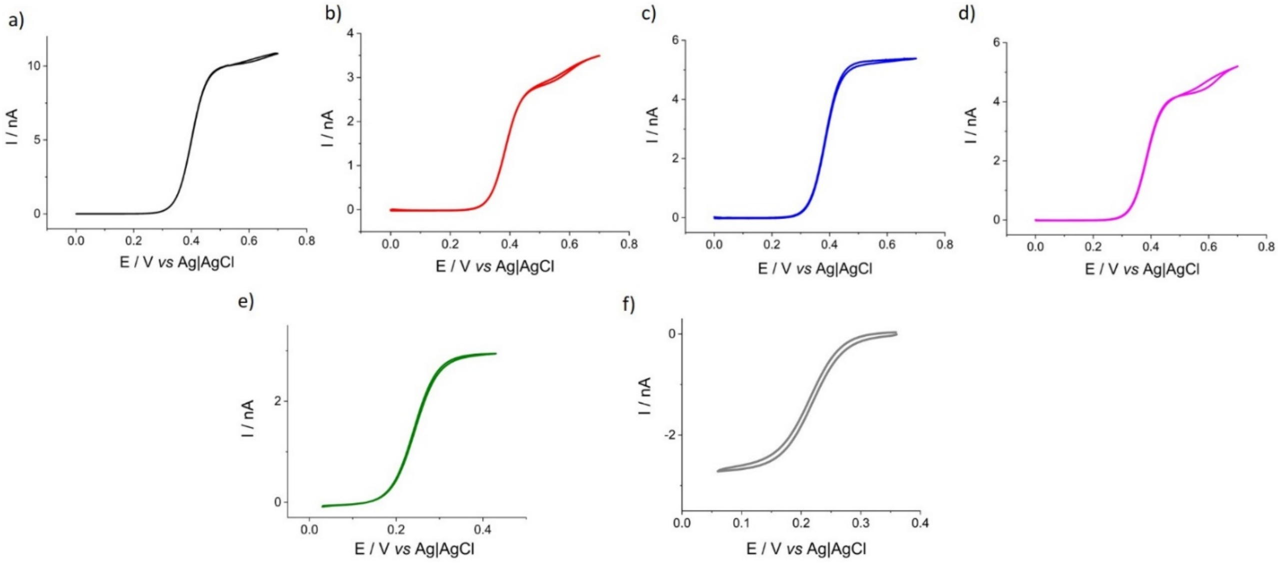

Table S1. Half-wave potentials of the redox mediators employed in the SECM measurements

Redox couple	$E_{1/2}$ vs. Ag|AgCl / V
Fc^+/Fc	+0.400

(*R*)-FcEA⁺/ (*R*)-FcEA	+0.395
(*S*)-FcEA⁺/ (*S*)-FcEA	+0.390
rac(±)-FcEA⁺/ *rac(±)*-FcEA	+0.395
$[FeCN)_6]^{3-/4-}$	+0.230

Figure S4 - Scheme of redox mediator regeneration at the substrate

The substrate under investigation is immersed in a solution containing the electroactive specie *R*, which can undergo the following reversible redox reaction:

$$R \rightleftharpoons O + e^-$$

At the SECM tip, a high positive potential is applied (i.e., more positive to the the halfwave potential, $E_{1/2}$, of the *O*/*R* couple), such that *R* is oxidised to *O* at a diffusion control rate (see Scheme S4a)

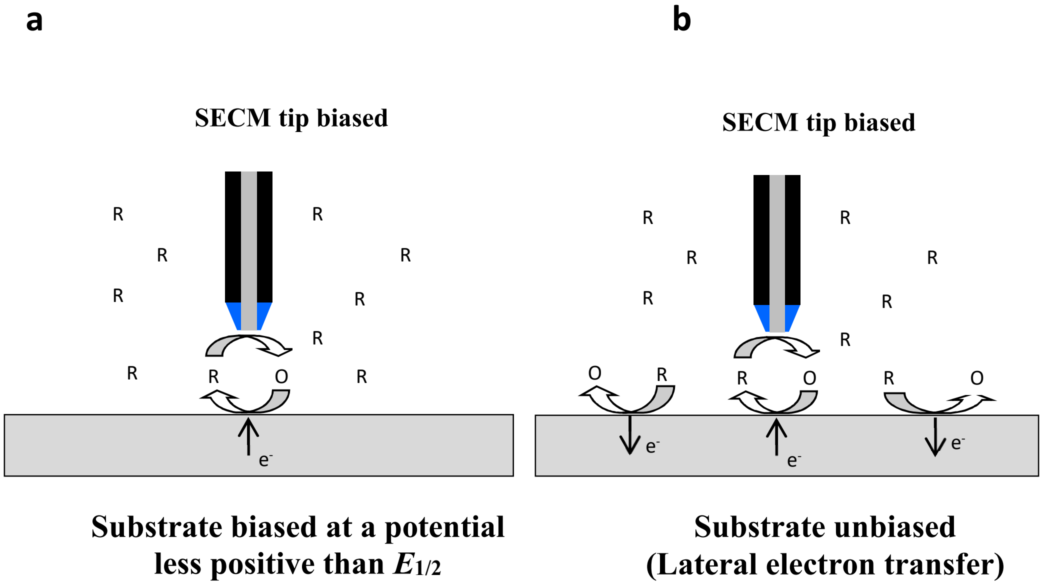

The redox mediator regeneration can also occurs at the unbiased substrate (Scheme S4b). This is because a significant area of the substrate below the tip, and away from it, is bathed by the solution whose concentration has not been perturbed by the tip-reaction [1-6]. Thus, in the latter zone, the substrate is poised at a potential established by the species *R* in the bulk solution and, at open circuit, its value is negative to the halfwave potential ($E_{1/2}$) of the *O*/*R* couple. When the tip approaches the substrate, the portion of the substrate directly under the tip is exposed to the electrode reaction product, *O*, and at a more positive potential. In this case, the localized reduction current at the portion of the substrate under the tip is driven by the oxidation of *R* at those portions of substrate away from the tip region [6]. This provides a lateral electron transport through the conducting material.
It must be noted that the potential of an unbiased substrate is also perturbed by current flow, which is required for recycling the mediator [4]. Thus, the feedback response can also be perturbed by finite heterogeneous electron-transfer kinetics [4].

In general, the lateral electron transport of Scheme S4b is operative in a conductive substrate whose surface area is larger than that of the micro-tip counterpart. In this work, the latte condition is fulfilled in the SECM experiments performed only at the bare gold electrode. Apparently, it was not the case for the measurements performed at the oligo-(*S*)-BT_2T_4-Au, as discussed in the main manuscript.

S5. Estimate of the average roughness of the bare gold and oligo-(*S*)-BT_2T_4-Au surfaces by SECM

The surface roughness profiles of the bare gold and oligo-(*S*)-BT_2T_4-Au surfaces were evaluated by using the current oscillations obtained in the 1-D scans. The current values were preliminarily normalized for the bulk current obtained for the specific redox mediator employed. The arithmetic mean current value (R_I) measured along a line was evaluated:

$$R_I = \frac{1}{N} \Sigma_{i=1}^{N} |I_i|$$

The amplitude of the current oscillation between the maximum peak ($\max_i I_i$) and minimum valley ($\min_i I_i$) were also evaluated. The maximum peak to valley height (R_{Imax}) was then established:

$$R_{Imax} = \max_i I_i - \min_i I_i$$

The values thus established were interpolated to the theoretical approach curves to transform current in normalized distance, *d*/*a*. By the knowledge of the microelectrode radius, roughness average (R_a) and maximum peak to valley height (R_{max}) were evaluated.

References

1. Bard, A.J.; Mirkin, M.V. (Eds.) *Scanning Electrochemical Microscopy*, 2nd ed.; CRC Press: Boca Raton, FL, USA, 2012.
2. Liljeroth, P.; Vanmaekelbergh, D.; Ruiz, V.; Konturri, K.; Jiang, H.; Kauppinen, E.; Quinn, B.M. Electron Transport in Two-Dimensional Arrays of Gold Nanocrystals

Investigated by Scanning Electrochemical Microscopy. *J. Am. Chem. Soc*. **2004**, *126*, 7126–7132.

3. Whitworth, A.L.; Mandler, D.; Unwin, P.R. Electron Transport in Two-Dimensional Arrays of Gold Nanocrystals Investigated by Scanning Electrochemical Microscopy *Phys. Chem. Chem. Phys*. **2005**, *7*, 356–365.
4. Wipf, D.O.; Bard, A.J. Scanning Electrochemical Microscopy: X. High Resolution Imaging of Active Sites on an Electrode Surface. *J. Electrochem. Soc.* **1991**, *138,* L4–L6.
5. Hocevar, S.B.; Daniele, S.; Bragato, C.; Ogorevc, B. Reactivity at the film/solution interface of ex situ prepared bismuth film electrodes: A scanning electrochemical microscopy (SECM) and atomic force microscopy (AFM) investigation. *Electrochim. Acta* **2007**, *53*, 555–560.
6. Bard, A.J.; Fan, Fu-Ren F.; Kwak, J.; Lev, O. Scanning Electrochemical Microscopy. Introduction and Principles. *Anal. Chem.* **1989**, *6*1, 132–138.
